# Reconstruction of Bilateral Upper and Lower Eyelid Ectropion Caused by a Liquid Unblocker Chemical Burn

**DOI:** 10.7759/cureus.40880

**Published:** 2023-06-24

**Authors:** Sophia Papadopoulou, Eirini Nikolaidou, Andrew P Joycey, Zoi Tzimorota, Eleni Karagergou

**Affiliations:** 1 Department of Burns, Plastic Surgery and Hand Surgery, General Hospital of Thessaloniki "G. Papanikolaou", Thessaloniki, GRC; 2 Department of Burns, Plastic Surgery and Hand Surgery, General Hospital of Thessaloniki “G. Papanikolaou”, Thessaloniki, GRC

**Keywords:** domestic accidents, drain unblockers, sulfuric acid burns, skin grafts, ectropion, eyelid burn, chemical burn

## Abstract

Liquid drain unblockers, although meant to be used by professionals with protective equipment, are sometimes used in the household without any precautions. This could lead to severe chemical burns, as in the case we present with severe eyelid ectropion. This study aims to stress the need for preventive measures regarding the use of chemicals and for close observation and timely surgical intervention in chemical burn patients to prevent and limit disfigurement.

A 45-year-old woman was injured while using an unblocker (90% sulfuric acid) at home. Accidentally, a quantity of the liquid was spilled on her face. She was initially examined in ophthalmology emergencies because of the obvious ocular involvement, and the cutaneous component was underestimated. On the third post-burn day, she was referred as an outpatient to our clinic, and because of the soft consistency and patchy pattern of the burn, she was asked to revisit in a week. Unfortunately, she reappeared two months post-burn with severe ectropion of all four eyelids and a high risk of corneal abrasion, desiccation, and further damage to the already injured left eye as well as the right eye. She underwent three operations in six months and a fourth 15 months after the accident, with the release of the scarred eyelids with full-thickness skin grafts, Z-plasties, and V-Y plasties. After four operations and sessions of triamcinolone acetonide intralesional injection, the patient has a satisfactory eyelid position and function with adequate closure and scar maturation.

Domestic use of strong industrial chemicals is dangerous, and public education for prevention is urgently needed. On the other hand, it is mandatory to follow up very closely with chemical burn patients to prevent severe sequelae, especially in the delicate and contraction-prone periocular and perioral areas. Reconstruction, in these cases, is a complex task. Often, several surgeries are needed to restore acceptable function and appearance. Burn disfigurement and self-stigma will follow the patients to some extent throughout their lives.

## Introduction

A wide variety of chemicals are commonly used in industry, agriculture, the household, and elsewhere. Many of them may cause cutaneous and ocular burns and systemic effects by either absorption or inhalation. This makes chemical burns an important risk in a household and the industrial setting. Although they only represent about 3% of all burns, they cause significant morbidity (nearly 55% of them require surgery), commonly involve critical areas like the face, thorax, and hands, and in some series, they induce approximately 30% of burn-related deaths [1-3].

Chemical injuries have some special features compared to thermal burns. They more likely result from longer exposure to chemicals, which may be continuing in the emergency room [2]. The extent of tissue damage is determined by the concentration and quantity of the chemical agent, duration of skin contact, penetration, and mechanism of action, and it may continue as long as traces of the offending agent are present [3].

Immediate removal of the involved clothing and thorough irrigation with water for periods of 30 minutes to two hours are the first important steps. Lavage dilutes and removes the chemical agent in contact with the skin. When the burn involves the eye, the current treatment recommendation is 0.9% saline decontamination to dilute and mechanically rinse the agent from the eye and eyelids and restore the pH to safe limits [3,4].

Drain openers are a common cause of household chemical burn injuries. They reportedly cause 56%-75% of domestic chemical injuries, either accidental or resulting from an assault [5].

We present a case of domestic chemical burn caused by the accidental splashing of a drain opener with a concentration of over 90% sulfuric acid over the patient's face.

The content of this article was previously presented as an oral presentation at the 19th European Burns Association Congress in September 2022 in Torino, Italy.

## Case presentation

**Figure 1:**
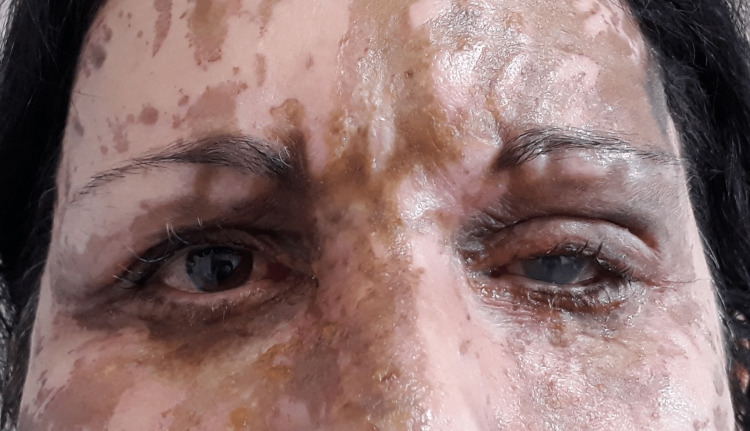
Facial burns on the third post-burn day

A 45-year-old previously healthy woman used a drain cleaner suggested by a neighbor as very "effective" to unblock a drain in her bathroom. Accidentally, the bottle slipped off her hands, and "a small" quantity of the liquid (sulfuric acid at 90%, as it proved to be) was spilled on her face. She reported excruciating pain immediately after the incident and washed her face with cold water for 15 minutes without making any specific effort to rinse her eyes. She initially visited the Ophthalmology emergency because of the obvious ocular involvement, especially of the left eye, while the cutaneous component was underestimated. She was referred to a specialized Ophthalmology Center in her hometown, and on the third post-burn day, she appeared on her initiative for consultation as an outpatient in our Burns Clinic. The corneal opacity of the left eye was obvious. She mentioned that the visual acuity of the left eye was estimated to be 2/10, and she received topical treatment with antibiotics, steroids, artificial tears, hyaluronic acid, and vitamin A. No formal referral or ocular burn grading from her attending Ophthalmologist was available. Due to the soft consistency and the marble cake-like burn pattern with normal tissue in between linearly burned skin, the patient received instructions for topical ointments, returned to ophthalmology care, and was asked to revisit in a week (Figure 1). Our first evaluation was of a mixed-depth burn, and it was obvious that surgical intervention would be needed.

**Figure 2:**
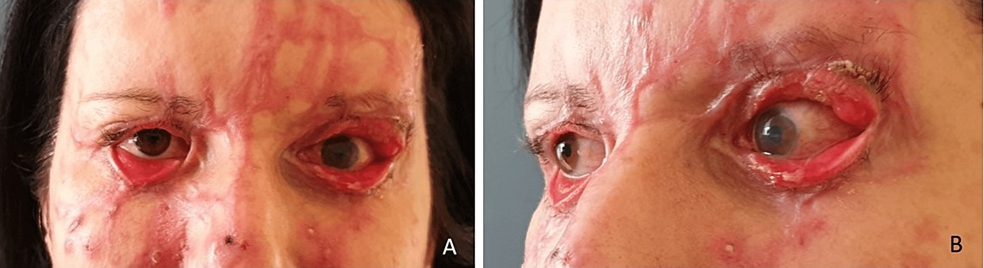
Ectropions two months post-burn (A) Eyelid burns two months post-burn. (B) Close-up of severe left upper and lower eyelid ectropions.

Unfortunately, she only reappeared in our clinic almost two months post-burn with severe cicatricial ectropions of all four eyelids with tarsal eversion. According to her excuses, the attending Ophthalmologist in her hometown would not refer her for eyelid surgery. On the other hand, this time period coincided with the onset of the COVID-19 pandemic and the first lockdowns, which made traveling between cities and accessing hospitals more difficult. The risk of corneal abrasion, desiccation, and further damage to the already injured left eye as well as the right eye was increased. Especially the left upper eyelid eyelashes were touching her eyebrow (Figure 2).

She consequently underwent three operations in three months and a fourth one 15 months after the accident, with the release of the scarred eyelids and full-thickness skin grafts (FTSG), Z-plasties, and V-Y plasties (Table 1).

**Table 1:** Timing of surgical procedures * FTSG: Full thickness skin graft

Procedure	Postburn-day
FTSG* left upper & lower eyelid, FTSG right lower eyelid, partial lateral tarsorrhaphy left	60
FTSG right upper eyelid	72
Repeat FTSG right lower eyelid	146
Tarsorrhaphy release with local flap, "Z" Plasties, "V-Y Plasties" both eyelids and canthal areas	474

**Figure 3:**
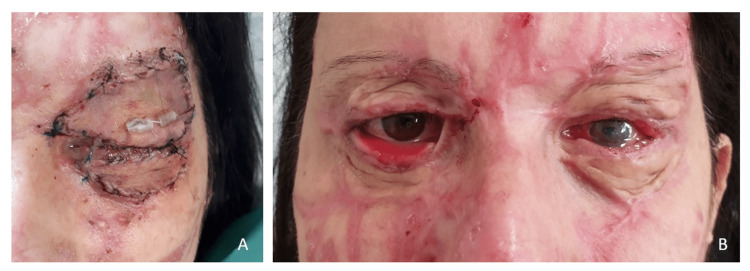
Skin grafts and recurrence A. Full thickness skin grafts on the left upper and lower eyelids, after the first operation B. Right lower eyelid ectropion recurrence

**Figure 4:**
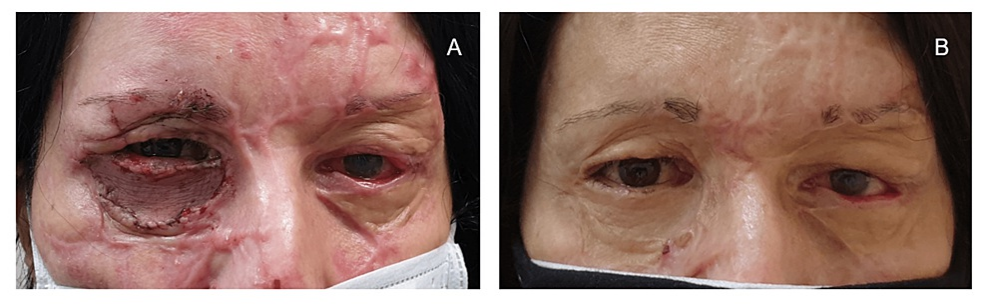
Early and one-year results after the third operation A. FTSG on the right lower eyelid, second time, early postoperative result. B. One-year postoperative result

We decided not to reconstruct all four eyelid ectropion simultaneously in the first operation because this would mean practically keeping both eyes closed for a week up to the graft check, an unacceptable situation for the already stressed and anxious patient. We chose to use full-thickness grafts to prevent graft contraction. The grafts were harvested from the inner arm and supraclavicular areas and secured with tie-over bolsters and Frost sutures [6,7]. Despite the attempted thorough scar releases beyond canthi and overcorrection, we did not avoid recurrence and reoperation (Figures 3, 4).

During the intervals between procedures, the patient was using a custom-made compressive face mask with silicone sheets in an attempt to keep scar tissue soft and flat. However, the scars kept contracting and pulling tissues even after many months.

Fifteen months post-burn, a fourth surgical procedure followed to release the lateral canthus of the left eye with a local flap as well as the web scars in the right medial canthus with Z-plasty and V-Y techniques. All these procedures were performed under general anesthesia since the patient would not accept surgery under local anesthesia or sedation. Between surgeries, sessions of triamcinolone acetonide intralesional injection were performed to soften specific areas with hypertrophic scarring.

**Figure 5:**
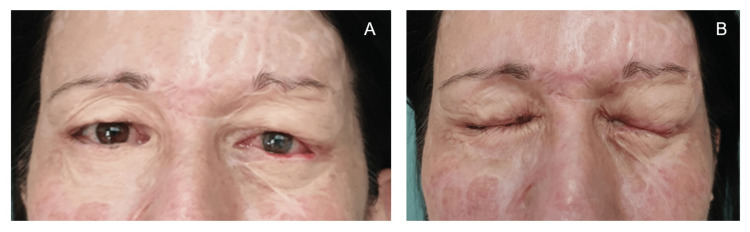
Postoperative result: 30 months postburn A. Situation 30 months postburn B. Satisfactory eyelid closure

Thirty months post-burn, the patient has satisfactory eyelid position and function with adequate unforced closure and scar maturation. Consequently, the ophthalmic condition also markedly improved, with a reduced need for lubrication and reaching a visual acuity of 7/10 for the threatened left eye. Still, there is room for improvement, especially with surgical refinements and methods such as laser treatments, nano fat injection techniques, or needling (Figure 5).

Throughout her treatment, serious appearance-related psychological distress, low self-esteem, and increased anxiety levels developed. Psychological consultation and support were needed. Meanwhile, she has achieved improvement and has returned to her pre-injury functional level and her work.

## Discussion

Chemical burns are uncommon (ca. 3%) but cause disproportionate morbidity, partly due to the ongoing activity of the chemical agent as long as it stays in contact with tissues [2,3]. The face is often involved (33%-58%) [1,5] in either assault or accidental chemical burns due to uncovered exposure. Even relatively small-area burns can cause serious sequelae due to the aesthetic and functional importance of the face [8]. The eyelids, with their major role of protecting the eye globe, have the thinnest skin (<1 mm) and are characterized by laxity and mobility. The skin sits on fat and muscle, so any deep burn tends to shrink with little resistance and is subjected to contraction forces towards the bony orbital margins, often leading to ectropion [8-10]. Accurate assessment of the burn depth and early intervention is essential to decide whether to excise and graft the wound early to prevent ectropion, corneal exposure, and ocular damage. Minor procedures like tarsorrhaphy or suspension sutures may also help limit the extent of retraction [10].

Unfortunately, estimating burn depth in chemical burns, especially on the first few days, is not straightforward. The appearance of an acute acid burn differs from that of a thermal burn; in an acid burn, the color ranges from light brown to black. Specifically, sulfuric acid burns cause brown, dark discoloration of the affected skin (Figure 1). The elasticity of the burned skin could help in burn depth assessment. A superficial and superficial partial-thickness burn, when palpated, resembles the texture of the patient’s adjacent normal skin; a full-thickness burn feels inelastic. However, a full-thickness eyelid acid burn may appear superficial with only mild discoloration of the skin and may be underestimated. Moreover, because of trickling, the acid wounds are often linear or patchy, with normal tissue in between burned skin, and difficult to manage surgically [1,3-5]. This was exactly the clinical picture of our patient at the first visit (Figure 1). Our first evaluation was of a mixed-depth burn, and it was obvious that surgical intervention would be needed. We decided, though, to reevaluate in a week to get a clearer picture. Assessment of burn depth is mainly critical for deciding whether to excise and graft the wound early.

There is no consensus over the optimal timing for excision and grafting of eyelid burns. The timing of surgery is often dictated by the emergence of eyelid contracture or ectropion when tissue destruction becomes demarcated, usually 2-3 weeks after injury [10,11]. As long as the eye is adequately covered, it is acceptable to wait for scar maturation for definitive correction [6]. Patients with eyelid burns should be examined daily, especially while asleep, to identify lagophthalmos, which can delay corneal healing [12]. The prevention of ectropion is critical. Most surgeons suggest proceeding with grafting as early as possible, but early grafting has been described as increasing complications, particularly infection, and increasing rates of recurrence and the need for reoperation. On the other hand, delayed skin grafting may increase the risk of hypertrophic scarring, asymmetry, and other deformities of the eyelid, leading to contractures and subsequent corneal exposure [4,10,12].

There is also controversy regarding the type of operation-scar excision or release and the type of graft used. In cases of early intervention, escharectomy and grafting are the obvious techniques applied, whereas, in cicatricial ectropion, it is important to release the eyelid scars as fully as possible to recreate the defect with overcorrection and dissection down to healthy tissue ‘canthus-to-canthus’ and extend the release up to 2 cm beyond the lateral canthus, angled upwards. It is suggested that this approach avoids damage to any remaining viable orbicularis oculi [6,10]. However, others consider scar excision of viable tissue essential to prevent further contracture while also providing a healthy wound bed for skin grafts take [4,10]. Full-thickness skin grafts (FTSG), usually from postauricular, supraclavicular, or inner arm areas, are preferred, although, in the past, it was suggested that thick split-thickness grafts (STSG) would be indicated for the upper eyelid to avoid excessive bulk [9,13]. Most authors nowadays agree on the use of FTSG over STSG to minimize the risk of graft contraction and ectropion recurrence since full-thickness grafts have much more dermis present and are subsequently less prone to contraction [6,10,11,13].

In our patient, the interruption of follow-up (the patient missed reexamination appointments for almost two months, having prioritized ophthalmologic treatment elsewhere) led to severe ectropion of all four eyelids, causing lagophthalmos and corneal irritation and complicating the healing process of the left corneal burn. Consequently, multiple releasing and grafting procedures, as well as minor local procedures, were needed primarily for functional and secondarily for the aesthetic reconstruction of eyelid scars. Additionally, serious appearance-related psychological distress, low self-concept and self-esteem, and increased anxiety levels were obvious in our patients. She avoided most social interactions and became depressed. Professional psychological consultation and support were needed and continued more than two years post-burn. Meanwhile, she has achieved improvement and has returned to her pre-injury functional level and her work.

The importance of close observation and follow-up of all chemical burn patients, as well as the coordination and teamwork of Plastic Surgeons and Ophthalmologists, which were missed in our case, in the assessment and treatment of periorbital and eyelid burns, cannot be overemphasized.

On the other hand, the serious hazards of household use of strong corrosive chemicals, such as drain openers, readily available on the market, must be pointed out. Despite warning signs on bottles, awareness of the potential harm of these agents is very low, and such chemicals can still be freely purchased and used by non-professionals at home without special protective measures. Burn prevention campaigns and public education programs are needed, and special regulations should be implemented to prevent these devastating and disfiguring injuries.

## Conclusions

Domestic use of strong industrial chemicals is dangerous. Restriction of the availability of certain high-concentration products, burn prevention programs, and the creation of public awareness can hopefully reduce accidental household chemical burn incidence. These burns do not usually involve a very large area but can cause significant morbidity. Our case illustrates the importance of a very close follow-up of the chemical burn victims to prevent severe sequelae, especially in the delicate and contraction-prone periocular area. Reconstruction, in these cases, is a complex task. Often, several surgeries are needed to restore acceptable post-injury function and appearance, a process that will go on as long as the patient seeks improvement while the sequelae follow the patient throughout their life.
